# MARDy 2.0: a platform for exploring and analysing antifungal resistance mutations

**DOI:** 10.1093/bioinformatics/btag786

**Published:** 2026-10-19

**Authors:** Joe Webber, Hugh Gifford, Johanna Rhodes, Rhys A Farrer

**Affiliations:** Medical Research Council Centre for Medical Mycology, University of Exeter, Exeter EX4 4QJ, United Kingdom; Medical Research Council Centre for Medical Mycology, University of Exeter, Exeter EX4 4QJ, United Kingdom; School of Biosciences, University of Birmingham, Birmingham B15 2TT, United Kingdom; Medical Research Council Centre for Medical Mycology, University of Exeter, Exeter EX4 4QJ, United Kingdom

## Abstract

**Motivation:**

The rising incidence of antifungal drug resistance represents a major and growing threat to global public health and food security. Therefore, understanding the genetic and molecular mechanisms that underpin antifungal resistance is essential for both clinical management and agricultural disease control.

**Results:**

We present MARDy 2.0, an updated platform replacing the original Mycology Antifungal Resistance Database. MARDy 2.0 provides an interactive platform for exploring antifungal resistance data from the community resource FungAMR, and protein structures from UniProtKB or predicted structures using AlphaFold. The platform provides enhanced tools for data exploration and analysis and is freely accessible via an interactive web interface that supports search filtering and downloads, BLAST-based sequence similarity searches, an experimental natural-language query interface for exploratory analysis, a mutation plot for visualizing mutations in a protein context, and structural predictions showing mutation locations. MARDy 2.0 provides a platform to facilitate research into antifungal resistance evolution, surveillance, and mitigation strategies. MARDy should be used and cited alongside the underlying resources it integrates, FungAMR.

**Availability and implementation:**

Accessible at https://mardy.net.

## 1 Introduction

Fungal infections are responsible for >1.5 million deaths annually, with antifungal drug resistance increasingly leading to treatment failure in clinical settings (Denning 2024). In parallel, the widespread use of fungicides in agriculture has driven the emergence of resistant fungal populations in the environment (Rhodes *et al.* 2022), resulting in crop protection failures and threatening global food security (Steinberg and Gurr 2020). These clinical and environmental reservoirs of resistance are increasingly recognized as interconnected, suggesting the need for integrated surveillance and data resources.

Recently, FungAMR (Bédard *et al.* 2025) has established a large-scale, community-driven resources for antifungal resistance mutations, providing a structured and openly accessible dataset that is actively maintained and expanded. This initiative represents a critical foundation for antifungal resistance research and enables downstream tools and platforms to build upon a shared, curated knowledge base.

The Mycology Antifungal Resistance Database (MARDy 1.0) is a platform that was originally developed to collate known genetic mechanisms of antifungal resistance across human, animal, and plant pathogenic fungi (Nash *et al.* 2018). This initial release contained 139 resistance mechanisms spanning 39 fungal species and 24 antifungal drugs and provided one of the first dedicated resources for fungal antimicrobial resistance research. MARDy subsequently served as the foundation for several related resources and analyses (Jain *et al.* 2023, Santana De Carvalho *et al.* 2024, Bédard *et al.* 2025, Sun *et al.* 2023, 2026), with portions of the FungAMR dataset now integrated into the Comprehensive Antibiotic Resistance Database (CARD; Alcock *et al.* 2023).

Since the release of MARDy 1.0, the volume of published data on antifungal resistance has expanded rapidly, driven by advances in genomics, functional genetics, and experimental evolution (Berman and Krysan 2020, Fisher *et al.* 2022, Vitiello *et al.* 2023, Brown *et al.* 2024). This has necessitated the development of a substantially expanded and modernized platform capable of supporting traditional lookup queries and large-scale exploratory analyses. MARDy 2.0 builds upon an established community resource, FungAMR, by providing a complementary platform focused on data interrogation, visualization, and analysis.

## 2 Materials and methods

### 2.1 Data collection and curation

MARDy 2.0 integrates resistance mechanism data from major community resources, most notably FungAMR (Bédard *et al.* 2025), which includes data from MARDy 1.0 (Nash *et al.* 2018); nonredundant data from CanDRes (Kumar *et al.* 2026); and peer-reviewed publications are also included, providing a web-based platform to access, visualize, and analyse the data. Entries from FungAMR were included if sufficient information was available to define (i) fungal species, (ii) antifungal compound, (iii) genetic resistance mechanism (e.g. amino acid substitution, insertion/deletion, gene overexpression), (iv) associated gene or protein, (v) host or usage context (clinical, agricultural, and experimental), and (vi) primary literature source (PubMed ID and/or DOI). Where multiple sequence accessions were available for a given entry, accessions from representative/type-strain annotated reference genome assemblies were selected to maximize consistency across studies.

Where reported, minimum inhibitory concentration (MIC) values, fold-change measurements, and experimental context were recorded. MIC values were normalized into structured fields (type, operator, minimum and maximum values, and units) and rendered into a single human-readable composite format for display. All entries were reviewed to ensure consistency in gene naming, species nomenclature, and drug identifiers. The current release of MARDy 2.0 provides access to 76 098 resistance-associated entries (covering point mutations, insertions, deletions, duplications, promoter tandem repeats, loss of function, repression, gene amplification/overexpression, and gene knockout) derived from 453 publications, covering 216 genes across 120 fungal species (Fig. 1A–C).

**Figure 1:**
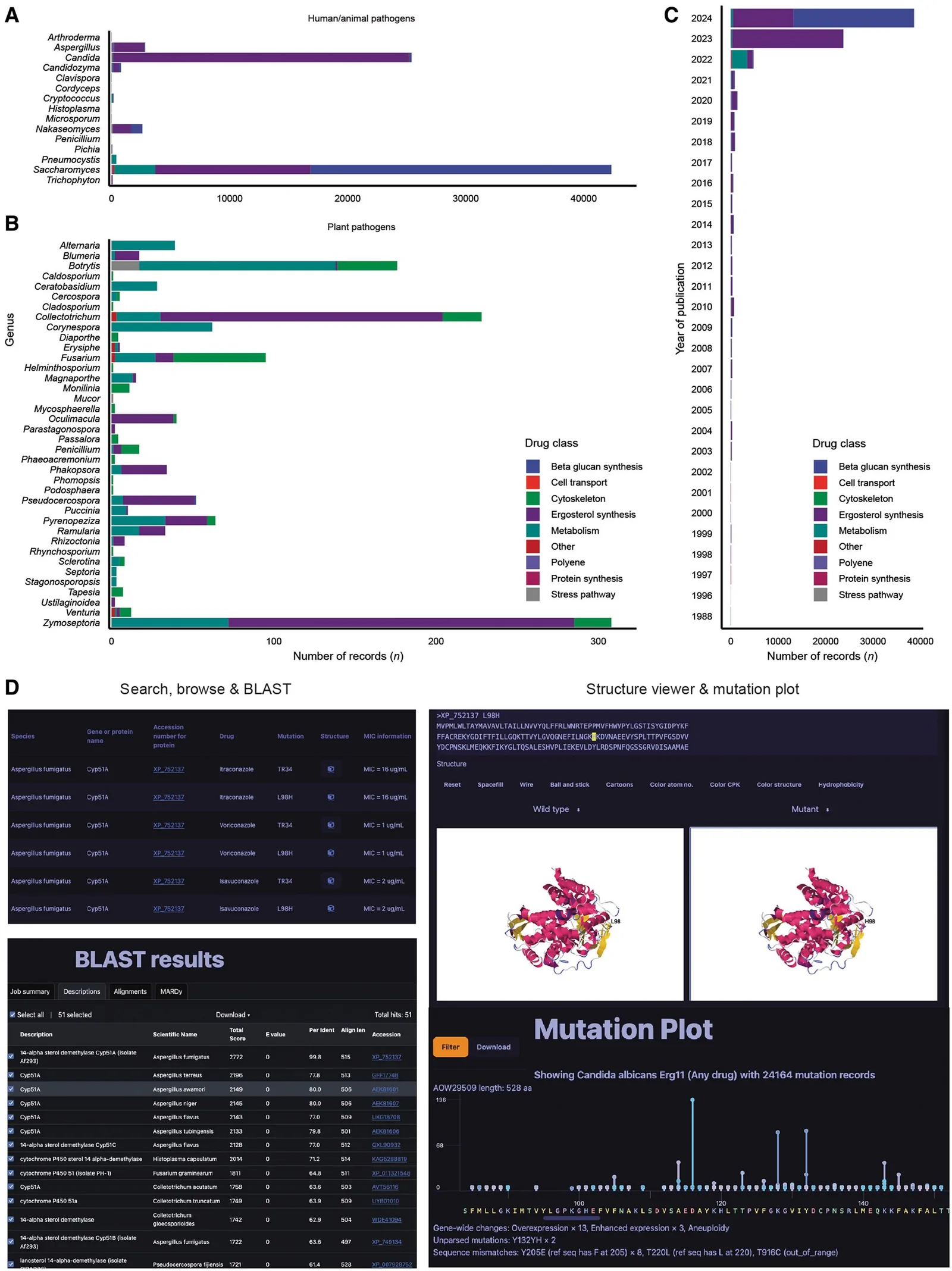
Database entries for (A) human and animal or (B) plant pathogens classified by Genus and coloured according to drug class. (C) Database entries coloured according to drug class, classified by year of publication. (D) Screenshots of mardy.net showing functionality, including searching and browsing the database, BLAST searches, a mutation plot for *Candida albicans* ERG11 and the structure for *Aspergillus fumigatus* Cyp51A (mutation L98H).

MARDy 2.0 integrates data from multiple community resources, including FungAMR, which is actively maintained and regularly updated. Users of MARDy should cite both MARDy and the underlying data resources, particularly FungAMR (Bédard *et al.* 2025), when using the platform for research or analysis. This ensures appropriate attribution to the original data generators and supports continued community-driven curation efforts.

### 2.2 Database architecture

MARDy 2.0 is implemented using a MySQL (version 8) relational database backend, with a schema optimized for flexible querying across species, genes, drugs, resistance mechanisms, and experimental context. Indexed fields support efficient filtering and sorting across large result sets, while derived fields (e.g. formatted MIC values) are precomputed to improve performance and usability. The web application is served via a PHP-based backend, which allows both HTML views and JSON APIs that are used by the interactive front-end. All data are freely accessible without registration, while optional user accounts enable additional functionality such as extended AI and BLAST search, and Mutation Plots. Registration will also allow future updates to be more easily communicated.

### 2.3 Web interface and search functionality

The MARDy 2.0 web interface provides multiple complementary modes of data access. Users can query the database using simple or advanced filters across species, genes, drugs, mutations, MIC values, host context, publication metadata, and additional annotations. Results are presented in a dynamically configurable table, allowing users to show or hide columns, sort by any field, and export subsets for downstream analysis.

To facilitate sequence-driven exploration, MARDy 2.0 integrates a BLAST (v. 2.16.0+) search interface (Altschul *et al.* 1990) that allows users to submit protein or nucleotide sequences and identify homologous resistance-associated genes within the database. BLAST results are directly linked back to curated resistance entries, enabling rapid contextualization of novel or uncharacterized variants. In addition, Mutation Plots are generated as a stand-alone webpage, which visualize the number of database records for any given species, protein, and drug, showing the amino acid location for those records. Clicking on a bar representing n number of records links to the relevant structure page to see the structural impact of those mutations.

MARDy 2.0 includes an experimental AI-powered query interface that allows users to pose free-text questions (e.g. “show only *Fusarium* resistance mechanisms” or “summarise *Candida* mutations since 2020”). The system interprets user intent, translates queries into structured database filters or aggregate analyses, and returns both tabular results and explanatory summaries. This feature is intended to support exploratory analysis and hypothesis generation, particularly for users unfamiliar with the underlying schema.

For each protein listed in the database, a dedicated structural page can be reached from either the database search or the mutation plot. Wild-type structures (PDB) were obtained from UniProtKB (Boutet *et al.* 2007). Where no experimental structure was available, models were generated using the AlphaFold Server (Abramson *et al.* 2024). AlphaFold model0 CIF files were converted to PDB using the Gemmi Python library (Wojdyr 2022). Mutant structures were generated using the PyMOL mutagenesis wizard (PyMOL Molecular Graphics System 2025). The structural visualization framework represents a distinct component of the MARDy platform, enabling users to contextualize resistance-associated mutations within experimentally determined or predicted protein structures.

## 3 Summary

MARDy 2.0 provides an open-access web-based platform for exploring, visualizing, and analysing antifungal resistance datasets, particularly FungAMR, through integrated search, sequence similarity analysis, mutation visualization, and structural interpretation tools. The platform is intended to complement existing community-maintained resources by providing enhanced functionality for exploratory analysis and data interrogation. MARDy 2.0 is freely available at https://mardy.net with no restrictions on access or use (Fig. 1D). Planned future developments include expanded structural coverage and integration with emerging surveillance datasets.
